# What can we learn from the evidence of psychosocial support for carers of people with cancer and how do we advance our efforts? A meta-review study

**DOI:** 10.1007/s11764-025-01802-8

**Published:** 2025-05-16

**Authors:** Brona Nic Giolla Easpaig, Bronwyn Newman, Judith Johnson, Rebekah Laidsaar-Powell, Ursula M. Sansom-Daly, Lucy Jones, Lukas Hofstätter, Eden G. Robertson, Stephen Mears, Kabir Sattarshetty, Rachel E. Houweling, Rhiannon Edge, Joanne Cummings, Reema Harrison

**Affiliations:** 1 School of Nursing, Charles Darwin University, Sydney, Australia; 2 Australian Institute for Health Innovation, Macquarie University, Sydney, Australia; 3 Division of Nursing, Midwifery and Social Work, School of Health Sciences, University of Manchester, Manchester, UK; 4 School of Psychology, University of Leeds, Leeds, UK; 5 Psycho-Oncology Co-Operative Group (PoCoG), School of Psychology, Faculty of Science, The University of Sydney, Sydney, Australia; 6 School of Clinical Medicine, Discipline of Paediatrics & Child Health, UNSW Medicine and Health, University of New South Wales, Sydney, Australia; 7 Behavioural Sciences Unit, Kids Cancer Centre, Sydney Children’S Hospital, Sydney, Australia; 8 Sydney Youth Cancer Service, Nelune Comprehensive Cancer Centre, Prince of Wales Hospital, Sydney, Australia; 9 Neuroblastoma Australia, Sydney, Australia; 10 Carers New South Wales, Sydney, Australia; 11 Redkite, Sydney, Australia; 12 Community First Step, Sydney, Australia

**Keywords:** Psychosocial intervention, Cancer, Carer, Caregiver, Support

## Abstract

**Purpose:**

Literature concerning programmes to support the well-being of carers of cancer survivors is vast, complex, and difficult to navigate, posing difficulties for identifying and translating relevant evidence. This study will advance the field by mapping and synthesising reviews to address the question: “What psychosocial interventions are available to promote the well-being of carers for people diagnosed with cancer, as reported in the evidence from reviews?”.

**Methods:**

A meta-review was conducted of reviews published between 2013 and 2024. PsycINFO, MEDLINE, CINAHL, and Cochrane Database of Systematic Reviews databases and relevant reference lists were searched for eligible reviews. Records were screened and assessed in accordance with the method with data from included reviews extracted and synthesised. The Preferred Reporting Items for Systematic Reviews and Meta-Analysis guided reporting. Reviews were appraised using the JBI Critical Appraisal Checklist for Systematic Reviews and Research Syntheses.

**Results:**

Ultimately, 54 reviews met the inclusion criteria. This evidence was mapped regarding populations, interventions, and outcomes, including depression, anxiety, quality of life, and distress. On average, reviews addressed nine of 11 quality checklist items. Insights were gained concerning the role of theory, carer populations, and intervention characteristics.

**Conclusion:**

Key features of the body of evidence were identified that can hinder progress and which point to ways forward. It is concluded that a recalibrated carer research agenda is needed, one that is designed to synthesise evidence concerning what works, for which carers to achieve what outcomes, or to address which kinds of psychosocial support needs.

**Implications for Cancer Survivors:**

Despite the need to ensure that carers have access to programs that support their wellbeing as they care for their loved ones, the evidence to guide program development is complex and difficult to navigate. A refocusing of research efforts is needed to advance understanding of what is most effective for carers, and how this can be translated into clinical practice.

**Supplementary Information:**

The online version contains supplementary material available at https://doi.org/10.1007/s11764-025-01802-8.

## Introduction

Carers for a person who has received a cancer diagnosis (i.e. inclusive of someone at any point in their journey from having received a diagnosis of and treatment for cancer) become core to healthcare teams [1, 2] and critical to strong healthcare partnerships [3]. The role of carers extends well into post-treatment survivorship and is particularly crucial for those who are living in the longer-term with advanced cancer. Carers may be family members and friends who assume responsibilities for the care and support of their loved one. Their work is vital to those who rely upon them, including in supporting survivors in making decisions about their care [4] and critical to the sustainability of the healthcare system [3, 5]. Relative to the general population, carers have poorer well-being and experience a high burden of mental health concerns, including distress, anxiety, and depression [6–10]. The distress associated with the illness of a family member or friend can be compounded by having caretaking responsibilities [11]. These responsibilities can be challenging and extensive: from the coordination and organisation of care, through to providing direct clinical care and facilitating lifestyle interventions [5, 12, 13]. Carers report feeling overwhelmed and underprepared in assuming these roles [14]. Further, distress experienced by survivors and carers may be interrelated, with the bi-directional impacts of stress noted in the literature [15].

It is recommended that models of survivorship account for the well-being of carers and encourage the attainment of necessary support [15–17]. Psychosocial interventions provide a significant opportunity to mitigate these challenges and support carer well-being [6, 18]. Understanding the evidence base concerning the available psychosocial support interventions for this group is particularly timely given recent Government focus on developing a strategy to best support carers as a matter of priority [19–21]. To address this clinical and policy imperative, comprehensive and systematic reviews that further the understanding of their unique set of needs and challenges are needed.

The evidence base concerning psychosocial interventions for carers appears to have grown exponentially, both in relevant primary intervention studies as well as reviews of these studies [14, 22, 23]. While the increased reporting of efforts to identify effective supportive programmes is encouraging, there remain significant shortfalls in understanding what is most effective for carers, and how this can be translated into clinical practice [24]. In a preliminary exploration of the literature, several challenges for navigating and making use of this evidence were apparent. First, efforts to develop psychosocial programmes to support carer well-being have been undertaken from a wide range of perspectives, for varied end-users, and with varying primary outcomes. While this has resulted in a large number of interventions, this breadth and the heterogeneity of interventions have yielded a disconnected evidence base and hindered the ability to advance carer support as a research community.

Second, carers may not necessarily be the primary target population for interventions [25]. Indeed, some ‘carer’ interventions were developed for survivors and subsequently extended to include carers. Further, in some cases, reviews of intervention studies have reported a combination of both the carer and survivor outcomes (e.g. Ownsworth, et al., 2021) [26]. This is a potential limitation of the literature as it can be challenging to disentangle the carer-specific outcomes associated with these interventions.

Third, collectively this body of work is highly diverse and even fragmented. There are important dimensions of differences such as in the target populations (e.g. dyadic, couples-focused vs. for parents of paediatric surivors) and the nature of interventions (self-guided app- based vs. in person group-based therapeutic programme) [27]. The divergences in the scope and focus of research comprising this evidence base can render it difficult to determine the evidence most relevant in the development and planning of a particular service.

As an initial step to help address these challenges, a meta-review will be undertaken in an attempt to develop a picture of the landscape of support for carers of people diagnosed with cancer [28, 29]. Meta-reviews in related areas (e.g. family-focused programmes in for supporting someone receiving palliative cancer care and interventions for carers of people experiencing a wide range of conditions) [30, 31] have proved highly valuable. The meta-review method was designed to facilitate a mapping of the evidence with attention to important dimensions such as populations and intervention characteristics [32, 33]. Moreover, this approach can help identify critical gaps in our current knowledge and enables the formulation of recommendations to guide future research efforts.

This meta-review aimed to address the following research question: “What psychosocial interventions are available to promote the well-being of carers of people diagnosed with cancer, as reported in the evidence from reviews?”.

The specific aims of the meta-review were to:

- Identify and synthesise findings of reviews reporting on findings from research concerning psychosocial interventions for caregivers of people diagnosed with cancer
- Critically appraise the selected review articles
- Formulate recommendations to advance the evidence base

## Method

An established meta-review methodology guided this study [34]. The Preferred Reporting Items for Systematic Reviews and Meta-Analysis (PRISMA) 2020 provided the reporting framework [35]. The protocol was registered with the International Prospective Register of Systematic Reviews (reference: CRD42023403219) and was published [36].

### Eligibility criteria for included reviews

#### Inclusion criteria

##### Population

- Carer of a family member (adult or child) or a person with whom they have a personal relationship who has received a diagnosis of cancer.
- People who are providing care on an ‘informal’ basis, which they have not been professionally contracted to provide following a cancer diagnosis and might involve care during treatments and after treatments are completed.

##### Intervention

- Non‐pharmacological interventions which are designed to positively impact some aspect of the psychosocial well-being of carers of people diagnosed with cancer (such as interventions focused on improving mental health).
- Psychosocial interventions can include educational, informational, and therapeutic activities designed to promote well-being (e.g. cognitive behavioural therapy).
- Remote, in-person and hybrid modes of intervention delivery were eligible for inclusion.
- Facilitated (e.g. by a health professional) and self-directed interventions delivered in any setting were eligible.

##### Outcomes

Reviews were included which had a primary focus and reported on the psychosocial outcomes of carers (e.g. quality of life).

##### Study methods

- Any type of systematically conducted review which detailed an established review method (e.g. systematic reviews, meta-reviews)
- Reviews of studies using any methods were eligible.

##### Publications

Review studies published in English between January 2013 and March 2024 in peer reviewed outlets were included.

#### Exclusion criteria

Reviews were excluded where psychosocial well-being was not a stated primary outcome of interest, carers were not the sole or primary focus, or the results of carers were not reported separately from other results. Additionally, conference abstracts, editorials, opinion pieces, published theses, and non-peer reviewed research were excluded.

### Search strategy

The approach employed in a recent relevant Cochrane systematic review [37] alongside a conceptual model of the cancer family carer experience [38] formed the basis of this search. Search strategy development was undertaken with the expertise of a medical research librarian (see Supplementary file 1 for list of terms). PsycINFO, Medline, CINAHL, and Cochrane Database of Systematic Reviews were initially searched for the period of between the 1 st of January 2013 until the 1 st of January 2023 and the search updated on the 28 th of March, 2024. The selected timeframe was considered to capture of a substantial period in which contemporary review evidence was generated.

### Review screening and selection

Returned records were imported into the Covidence platform [39] and deduplicated. Each title and abstract were screened for eligibility by a team member (BNGE, BN, RH, REH, USD, KS, RE, JC) and were included; the full text then assessed for inclusion by two team members (BNGE, BN, RH, REH) independently. Discrepancies were resolved either via team discussion or by a third reviewer. The reasons for exclusion of full texts were recorded.

### Critical appraisal

A team member (BNGE) undertook appraisal of the quality of methodological reporting using the JBI Critical Appraisal Checklist for Systematic Reviews and Research Syntheses [40] with another team member (RH, BN, RLP, KS, REH) independently examining the texts and reviewing the recorded appraisal. Discrepancies were resolved via team discussion.

### Data collection and synthesis

A data extraction template generated using the Microsoft Excel application [41] was piloted by two team members (BNGE, KS) who independently extracted data for a cross section of studies (10%). The remainder of the data were extracted by a team member (BNGE) and then reviewed independently by another (RH, BN, RLP, REH) with inconsistencies resolved through discussion. Data items included study characteristics (e.g. review aims), populations (e.g. target carer population), intervention details (e.g. mode of delivery), theoretical bases, and outcomes of interest (e.g. depression outcomes). Extracted data were synthesised and results presented in a narrative description [34].

## Results

**Fig. 1:**
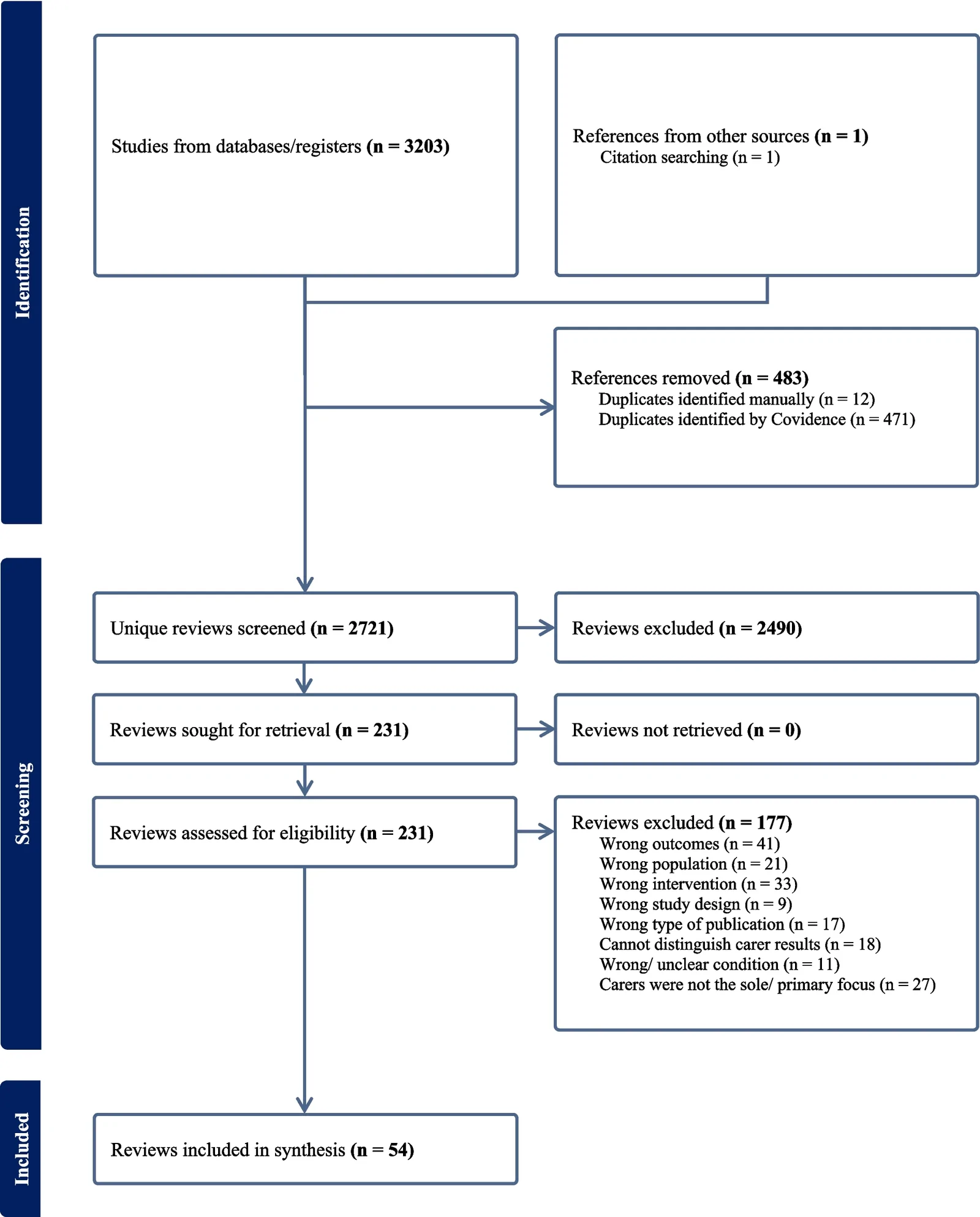
Meta-review results as shown in PRISMA flow diagram (2020)

Following the study selection, 54 reviews were deemed eligible for inclusion [12, 14, 22, 23, 30, 31, 37, 42–88] (see Fig. 1). The number of relevant publications appears to have been growing, the majority of which were systematic reviews (*n* = 46), with meta-analysis undertaken in 20/54 review (see Table 1 for details). Critical appraisal showed that on average, nine of the 11 checklist quality items were addressed in reviews (ranging from three to 11) with the lowest scoring items concerning assessment of publication bias and the independent conduct of critical appraisal (see Supplementary file 2 for further details). Please note that certain items are not necessarily applicable to particular review methods.

**Table 1:** Key study characteristics of included reviews

Citation	Year	Review aim(s) (verbatim from abstract)	Review type	Condition(s) of those cared for	Study types/designs included in review	Search period	Details of intervention population	Patient population: adult/child
Ahn et al	2020	To examine the characteristics of interventions to support family caregivers of patients with advanced cancer	Systematic review	Advanced cancer	RCTs and quasi-experimental designs	2007–2018	Family caregivers of adult patients with advanced cancer	Adult
Akechi et al	2022	This study initially began as part of a research study on the development of effective psychological care for bereaved family members of cancer patients and was supported by a Grant-in-Aid for Cancer Research from the Japanese Ministry of Labour, Health, and Welfare, followed by academic support from the Japanese Psycho-Oncology Society (JPOS) and the Japanese Association of Supportive Care in Cancer. In this article, we describe the process involved in developing these guidelines and present a summary of the recommendations for the psychological care of bereaved families who have lost a member to a physical illness	Systematic review	Physical illness including cancer	RCTs	Inception–2020	Bereaved families of patients with a physical illness including cancer	Both
Al Daken et al	2018	This review aims to determine the effectiveness of mindfulness-based interventions (MBIs) and educational interventions (EIs) as supportive care for family caregivers (FCs) of patients with cancer	Systematic review	Cancer (any)	RCTs and quasi-experimental studies	2007–2017	Family carers of people with cancer and or patients with cancer receiving palliative care	Not specified/unclear
Applebaum et al	2013	The purpose of the present study was to characterise the state of the science of psychosocial interventions for informal cancer caregivers	Systematic review	Cancer (any)	RCTs, as well as interventions not yet at the RCT level (i.e. pilot studies)	1980–2011	Informal carers of a person with cancer	Not specified/unclear
Areia et al	2020	The purpose of this study is to review the current state of the art in family/caregiver-focused interventions of people with terminal cancer in palliative care	Meta review	Advanced cancer	Systematic reviews of RCTs	2008–2018	Family/caregiver focused interventions of people with terminal cancer in palliative care	Adult
Bautista et al	2021	This study provides a systematic review and meta-analysis of randomised controlled trials that analysed the efficacy of psychological interventions for parents of children with cancer	Systematic review	Cancer (any) children	RCTs	Inception–2017	Parents of children with cancer	Paediatric
Becqué et al	2023	Therefore, we aimed to (1) systematically examine characteristics of supportive interventions for family caregivers of patients with advanced cancer, (2) systematically examine the evidence on the effectiveness of these interventions on different outcome dimensions, and (3) assess whether characteristics of supportive interventions are associated with family caregivers’ outcomes	Systematic review	Advanced cancer	Quantitative studies	2004–2020	Adult family caregivers providing care to patients with advanced cancer	Not specified/unclear
Boele et al	2019	To assess the effectiveness of supportive interventions at improving the well-being of caregivers of people with a brain or spinal cord tumour. To assess the effects of supportive interventions for caregivers in improving the physical and emotional well-being of people with a brain or spinal cord tumour and to evaluate the health economic benefits of supportive interventions for caregivers	Systematic review	Neuro-oncology (over 20% of sample)	RCTs	Inception–2018	Adult caregivers (aged 18 years or older) for people with a brain or spinal cord tumour	Both
Cheng et al	2022	To evaluate the effectiveness of psychoeducational interventions on the outcomes of caregivers of patients with cancer and identify characteristics of effective psychoeducational interventions	Systematic review	Cancer (any)	RCTs	Inception–2020	Adult caregivers caring for adult patients with cancer	Adult
Chow et al	2023	We examined the effectiveness of interventions offering support for caregivers of patients with advanced cancer on caregiver QOL and mental health outcomes	Systematic review	Advanced cancer	RCTs	Inception–2021	Adult caregivers of adult patients with advanced (stage III or IV) cancer	Adult
Christodoulou et al	2024	This systematic review aims to examine the characteristics of psychological interventions that aim to enhance positive outcomes, such as well-being, post-traumatic growth, or hope in cancer caregivers	Systematic review	Cancer (any)	All study designs reporting primary data	Inception–2023	Adult unpaid caregivers of adult cancer patients	Adult
Dhumal et al	2023	Thus, the purpose of the current study was to conduct a systematic review with meta-analysis of RCTs to evaluate interventions aimed at reducing IC (informal caregivers) distress and burden among those caring for patients with cancer	Systematic review	Cancer (any)	RCTs	Inception–2021	Adult ICs engaged in provision of adult cancer care	Adult
Eccleston, et al	2015	To evaluate the efficacy of psychological therapies that include parents of children and adolescents with chronic illnesses including painful conditions, cancer, diabetes mellitus, asthma, traumatic brain injury (TBI), inflammatory bowel diseases (IBD), skin diseases, or gynaecological disorders. We also aimed to evaluate the adverse events related to implementation of psychological therapies for this population. Secondly, we aimed to evaluate the risk of bias of included studies and the quality of outcomes using the GRADE assessment	Systematic review	Chronic illness	RCTs	Inception–2014	Parents of children and adolescents with a chronic illness	Paediatric
Eche et al	2021	We conducted (i) a systematic review on psychosocial interventions for parents, with an exploratory focus on racial and ethnic minorities, and (ii) a meta-analysis to quantify the efficacy of existing interventions	Systematic review	Cancer (any) children	Prospective, quasi-experimental or experimental design	2000–2020	Parents of children with cancer	Paediatric
Ferrell & Wittenberg	2017	The purpose of this report is to present results of a review of the literature published since the previous meta-analysis for the period from 2010 through 2016	Systematic review	Cancer (any)	RCTs	2010–2016	Caregivers of adult patients with cancer	Adult
Frambes et al	2018	The purpose of this review was to analyse recent (2009–2016) caregiving literature for evidence of relationships between supportive interventions for caregivers of cancer patients, the care activities performed, caregiver characteristics, and resulting caregiver outcomes	Systematic review	Cancer (any)	RCTs	2009–2016	Caregivers of people with cancer	Not specified/unclear
Fu et al	2017	To systematically review the effect of psychosocial interventions on improving QoL [quality of life], depression, and anxiety of cancer caregivers	Systematic review	Cancer (any)	RCTs and quasi-experimental	2011–2016	Carers for people with cancer	Not specified/unclear
Gabriel et al	2020	To review the characteristics and effectiveness of psychosocial interventions on quality of life of adult people with cancer and their family caregivers	Systematic review	Cancer (any)	Intervention studies: randomised and non-randomised	2009–2019	Adult people with cancer and their family caregivers	Adult
Geertz et al	2023	This review aims to summarise peer-group interventions for children and adolescents of cancer patients, where those affected have the opportunity to exchange and normalise their feelings among equals	Systematic review	Cancer (any)	Evaluation or intervention studies	Inception–2022	Children and adolescents with a parent who has cancer or who has died of cancer	Adult
Heckel, et al	2019	The aims of this systematic review were to summarise the profile of caregivers accessing cancer helplines, to evaluate caregiver satisfaction with the helpline service, and to review the evidence base of intervention studies testing the efficacy of community-based cancer helplines in improving caregiver health and well-being	Systematic review	Cancer (any)	No restrictions	Inception–2018	Adult caregivers of adult cancer patients (any cancer type or disease stage)	Adult
Heinsch et al	2022	Expanding on the limited work in supportive care for friends and family caregivers of adults with a primary brain tumour, this review sought to examine all available evidence since 2010 on the efficacy and feasibility of supportive interventions for this population including non-controlled studies	Systematic review	Primary brain tumour	Quantitative, qualitative or mixed methods	2010–2021	Adult carer and patient cohort (> 18 years)	Adult
Hughes et al	2023	We aim to provide the first systematic review of the literature to date on skill building interventions for informal caregivers of adults with cancer. We also review the types of methodologies and outcomes used to determine effectiveness	Systematic review	Cancer (any)	No restrictions	Inception–2022	Informal caregivers of adults with cancer	Adult
Jones et al	2022	To systematically review and examine current evidence for the carer-reported benefits of supportive care strategies for carers of adults with high-grade glioma	Systematic review	High-grade glioma	No restriction stated: non-randomised studies, randomised trials, mixed-methods, qualitative, and quantitative descriptive study	2005–2022	Adult carers of adults with HGG	Adult
Kajiwara et al	2023	To identify nursing support for caregiver burden in family caregivers of patients with cancer	Scoping review	Cancer (any)	Studies with a quantitative outcome	Inception–2023	Caregivers of patients with cancer aged 18 years or older	Adult
Kaltenbaugh et al	2015	To explore the literature to examine the physical, social, psychological, financial, usability, and feasibility outcomes of web-based interventions on caregivers of people with cancer and to identify potential trends in this body of evidence	Systematic review	Cancer (any)	Quantitative	Inception–2014	Caregivers of adult patients with cancer aged 18 years or older	Adult
Kalyani et al	2023	The goal of this meta-analysis was to check the effect of psychosocial interventions on cancer’s caregiver quality of life and check the impact of various psychological intervention programmes	Systematic review	Cancer (any)	RCTs	2006–2021	Caregivers providing care to adult cancer patients	Adult
Kedia, et al	2019	The purpose of this systematic review is to examine the content, delivery, and efficacy of psychosocial interventions targeting caregivers of lung cancer patients	Systematic review	Lung cancer	No restriction stated: non-randomised, randomised, and review designs	2009–2017	Caregivers of patients with lung cancer	Not specified/unclear
Kleine et al	2019	This review aimed to present the content and the effects of interventions delivered to caregiving partners of cancer patients on both partners and patients. Furthermore, we provide information about the acceptability of the interventions and study quality	Systematic review	Cancer (any)	Quantitative and mixed methods studies	Inception–2017	The participants were spouses or partners (married or unmarried) of cancer patients aged 18 years or older	Adult
Koumarianou et al	2021	Thus, the primary purpose of the present paper is to systematically evaluate the evidence of the effectiveness of psychosocial interventions targeting families during their child’s active cancer treatment and to make recommendations for the direction of future research	Systematic review	Cancer (any)	Randomised controlled trials and quasi-experimental studies	2009–2017	Families with children newly diagnosed aged between 7 and 12 years	Paediatric
Kusi et al	2023	The current review will address this gap by exploring the effectiveness of PEI and identify up‐to‐date evidence on intervention components that can aid in the design and optimization of PEI for caregivers of adult patients living with cancer	Systematic review	Cancer (any)	RCTs and non‐RCTs	Inception–2021	Adult caregivers providing care to adult cancer patients	Adult
Lally et al	2016	The purpose of this review was to identify and evaluate psychoeducational intervention studies addressing the psychological adjustment needs of family members, spouses, and friends (supporters) providing support to women with early-stage breast cancer with the goal of guiding future research and practice	Integrative review	Breast cancer (early stage)	RCTs and pre-post designs (including pilots)	2003–2014	Supporters of women (patient) with early stage (0–III) breast cancer who were from a western culture	Adult
Lang et al	2014	The objective of this review was to synthesise the best available evidence of the effects of art therapy for family caregivers of cancer patients	Systematic review	Cancer (any)	Experimental study designs including quasi-experimental (pre, post)	2003–2013	Adult family caregivers	Not specified/unclear
Law et al	2019	To evaluate the efficacy and adverse events of psychological therapies for parents of children and adolescents with a chronic illness	Systematic review	Chronic illness	RCTs	Inception–2018	Parents of children with cancer	Paediatric
Lee et al	2021	This systematic review aimed to synthesise the effectiveness of psychosocial interventions on caregivers of advanced cancer patients, in comparison with usual care, on caregivers’ quality of life (QoL), anxiety, and depression symptoms	Systematic review	Advanced cancer	RCTs		Informal caregivers for a persons with advanced cancer	Not specified/unclear
Lehto et al	2021	The purpose of this paper was to evaluate the state of the science regarding the use of natural environment interventions to support caregivers of cancer patients in the community	Scoping review	Cancer (any)	No restrictions	Inception–2021	Informal caregivers for people with cancer	Not specified/unclear
Lei et al	2023	A systematic review and meta-analysis was conducted to (1) evaluate the effect of non-pharmacological interventions on the outcomes of depression and anxiety for lung cancer patients’ informal caregivers and (2) compare the effects of interventions with differing characteristics (i.e. intervention types, mode of contact, and group versus individual delivery)	Systematic review	Lung cancer	Intervention studies	2010–2022	Lung cancer patients’ informal caregivers	Not specified/unclear
Li et al	2022	To assess the impact of e-Health interventions on the caregiver burden, depression, and quality of life of informal caregivers of cancer patients	Systematic review	Cancer (any)	RCTs	Inception–2021	Adult informal caregivers of patients with cancer	Adult
Liu et al	2023	The aim of this review was to determine whether the caregivers of breast cancer patients who undergo psychosocial interventions report improvements in quality of life (QOL), depression, and anxiety	Systematic review	Breast cancer	RCTs and quasi experimental studies	Inception–2022	Primary caregivers of patients with breast cancer	Not specified/unclear
Murfield et al	2019	To provide an overview of the current use of mindfulness- and compassion-based interventions with family carers of older adults, to aid primary healthcare practitioners in their decision-making around referral to wider healthcare services	Scoping review	Conditions impacting older people: dementia, cancer, amyotrophic lateral sclerosis, chronic conditions, cirrhosis, and Parkinson’s disease	Quantitative, qualitative, or mixed-method research designs	Inception–2019	Adult carers of an older adult family member, friend, or neighbour	Adult
Petersen	2020	The purpose of this scoping review was to examine the evidence regarding the value of spirituality/spiritual care in minimising the vulnerability of parents whose children were diagnosed with cancer and who faced the end of life	Scoping review	Advanced cancer	No restrictions	Inception–2018	Parents of children who were diagnosed with cancer (including bereaved parents)	Paediatric
Roderick et al	2024	The purpose of this systematic review was to investigate the psychological and biological effects of mind–body therapies for informal caregivers of individuals with cancer	Systematic review	Cancer (any)	RCTs	Inception–2010	Adult carers of individuals with cancer	Both
Sak-Dankosky et al	2022	The aim of this study was to determine what kind of psychosocial interventions aimed at improving the well-being of adult cancer patient caregivers were developed, and to describe the methodological characteristics and clinical effectiveness of the interventions which could be included in the nursing care plans	Systematic review	Cancer (any)	RCTs and quasi-experimental studies	2004–2019	Caregivers of adult patients with cancer	Adult
Sánchez-Egea et al	2019	The overall aim of the present work was to perform a systematic review and meta‐analysis of empirical evidence on the efficacy of psychological treatments applied to family members of children and adolescents with cancer	Systematic review	Cancer (any)	RCTs and uncontrolled trials	1980–2017	Family members of children with cancer	Paediatric
Secinti et al	2022	This meta-analytic review examined the efficacy of psychosocial interventions in reducing caregiver burden in caregivers of adults with cancer	Systematic review	Cancer (any)	RCTs	Inception–2022	Adult carers of adults with cancer—notable that 79% of carers were Caucasian	Adult
Shin et al	2018	This paper aims to review literature published on the support of cancer caregivers with health technology	Systematic review	Cancer (any)	No restrictions	2008–2017	Caregivers of patents with cancer	Not specified/unclear
Sutanto et al	2017	To explore available psychosocial interventions for informal caregivers of palliative cancer patients	Scoping review	Advanced cancer	No restrictions		Adult family caregivers of adult palliative care patients	Adult
Tang et al	2020	This meta-analysis aims to evaluate the best available evidence concerning the effects of psychoeducational interventions (PEIs) on the psychosocial and coping outcomes for caregivers of paediatric oncology patients	Systematic review	Cancer (any)	RCTs	Inception–2017	Caregivers of children (up to 18 years) undergoing treatment for cancer	Paediatric
Thomas et al	2017	To update what is known about effective interventions to support carers of ill, disabled or older adults	Meta review	Illness, disability or older (including cancer)	Systematic reviews and meta-analyses	2009–2016	Carers of all ages (including children and young adults) supporting any adults (aged ≥ 18 years), including those with dementia, learning disabilities, and mental health problems	Adult
Treanor et al	2019	To assess the effectiveness of psychosocial interventions designed to improve the quality of life (QoL), physical health, and well-being of informal caregivers of people living with cancer compared with usual care	Systematic review	Cancer (any)	RCTs	Inception–2018	Adult non-professional caregiver of an adult patient with cancer	Adult
Waldron et al	2013	To evaluate and estimate the effect of psychosocial interventions on improving the quality of life (QoL) of adult cancer caregivers	Systematic review	Cancer (any)	RCTs	1947 to 2011	Cancer patient and caregiver (adults)	Adult
Winter et al	2023	The objective of this study was to summarise the literature on current interventions available for carers of men with prostate cancer and analyse the outcomes of these interventions in supporting carers’ needs	Systematic review	Prostate cancer	RCTs and pilot studies of interventions	Inception to 2022	Partners and informal carers of men with prostate cancer	Adult
Wootten et al	2014	The aim of this review is to summarise and critically evaluate the current literature reporting psychosocial intervention studies for partners of prostate cancer patients	Systematic review	Prostate cancer	Primary studies reporting on interventions (except descriptive)	Inception–2013	Partners of prostate cancer patients	Adult
Yıldız et al	2024	The purpose of this systematic review is to examine evidence-based psychosocial intervention research aimed at family members caring for patients with cancer in the palliative period	Systematic review	Advanced cancer	RCTs	2016–2021	Family members caring for patients with cancer	Not specified/unclear
Zhu et al	2024	To assess the effectiveness of web-based compassion interventions on the mental well-being of family caregivers	Systematic review	Any illness	RCTs	Unclear–possibly inception–manuscript submission date	Informal caregivers aged 18 years	Not specified/unclear

Only one review focused on children/young people caring for parents [56]. Patient populations cared for were adults (*n* = 29); paediatric; both paediatric and adult patient populations (*n* = 3), with this distinction unclear for the remainder of reviews (*n* = 17). Most reviews did not limit their focus to a specific type of cancer (*n* = 32). Where restricted, reviews concerned: advanced cancers (*n* = 8), neuro-oncological (*n* = 3); breast cancer (*n* = 2); prostate cancer (*n* = 2) and lung cancer (*n* = 1). The remaining studies included carers of people with cancer as part of carer groups for people with a range of illnesses (e.g. chronic conditions) (*n* = 6).

### Interventions

Interventions commonly included cognitive behavioural therapy, stress management, counselling and coping skills development; however, the presentation of details reported in reviews about the nature of intervention types was not sufficiently consistent or homogenous to allow categorisation. While in some reviews reference was made to the involvement of carers and other stakeholders in the development of interventions [37, 42, 82, 85], there was little information about this across included reviews.

In 23 reviews, the home was the sole, or one of the intervention settings, while community and medical facilities were also identified as settings. In 25/54 reviews, intervention settings were not reported, or were unclear. Facilitators of intervention delivery represented in reviews were commonly nurses (*n* = 28), mental health professionals (*n* = 24), social workers (*n* = 16), and doctors (*n* = 13) in addition to others. In 20 reviews, interventions were included which were wholly or in part self-guided. These details were not reported or were unclear in 16 reviews.

Most reviews included interventions delivered both in-person and remotely (30/54), remotely (*n* = 3) or in-person (*n* = 2) (information was not discernible for 19 reviews). Telephone contact (*n* = 34 reviews) and internet-based engagement (*n* = 32 reviews) were popular components. Half of reviews reported some information about the theoretical bases (encompassing concepts, models, frameworks, or specific theories that informed interventions) employed in some or all the studies included in the reviews (*n* = 28) (please see Supplementary file 3 for further details). While the importance of theory was noted in some reviews [55, 77], generally analysis of specific theories/theoretical components in relation to outcomes was not well-represented overall in reviews. To facilitate synthesis, theory data groupings are shown in Table 2.

**Table 2:** Overview of the groupings of theoretical bases represented in the included reviews

Theoretical groupings represented in reviews	Concepts, frameworks and bases included
Cognitive theories (*n* = 18 reviews)	Cognitive behavioural theory; socio-cognitive: social cognitive theory, cognitive/social processing, cognitive and social processing theories of adaptation to difficult life events and social learning; and efficacy/determination: self-efficacy and self-determination theory
Coping and stress theories (*n* = 17 reviews)	Stress and coping: stress-appraisal, creativity, optimism, planning, expert information framework, transaction model of stress and coping, meaning-based coping models, social cognitive processing model of emotional adjustment to cancer
Interpersonal/relationship-focused (*n* = 16 reviews)	Dyadic models, attachment theory, theoretical model of sexual recovery, a model of couples’ adaptation to illness, a theory of healthy relationship functioning, social support and interpersonal theories, sex therapy, and interdependence theory, and a communal coping approach, marital therapy, marital communication and psychosexual function, adaptation model of couples functioning, relationship intimacy model, family theory, family systems, family identity theory, family systems theory, and family-centred care
Specific therapies identified as theories (*n* = 6 reviews)	Cognitive behavioural therapy; existential behavioural therapy, emotional focused therapy; needs assessment tool-carers-guided consultations, art as support and adventure-based approach, and natural environment approaches
Change theories (*n* = 4 reviews)	Transition theory and adaptation theories
Problem solving/skills (*n* = 4 reviews)	Problem-solving/training framework, skills training, and social problem-solving model
Illness-focused theories (*n* = 3 reviews)	Wagner’s chronic illness care model and the illness uncertainty framework
Educational (*n* = 3 reviews)	Psycho-educational and supportive-educative
Grouped as ‘other’ (*n* = 14 reviews)	Quality-of-life frameworks, crisis intervention model, equity theory, positive psychology theory, bio-psychosocial model, symbolic relational model, relational framework theory, framework of dialectical philosophy and validation, mindfulness, and spirituality

### Outcomes of interventions reported

Depression, anxiety, quality of life and psychological distress outcomes were the most reported (other outcomes, such as caregiver burden were less frequently presented). Table 3 shows the overall appraisal for these commonly reported outcomes. The substantial heterogeneity of methods, operational definitions and measures used across reviews, limits the ability to contrast and compare findings between reviews.

**Table 3:** Summary of the number of reviews reporting findings for key outcomes

Outcome type	Improved	No significant change	Worsened	Unclear*	Not reported/insufficient detail +
Depressive symptoms	11	4	0	10	29
Anxiety	9	5	0	9	31
Quality of life	12	3	0	12	27
Psychological distress	7	2	0	5	40

* ‘Unclear’ was given where there were mixed findings or significant uncertainty (e.g. where authors expressed that a conclusion could not be reliably reached)

+ ‘Not reported/insufficient detail’ captures instances in which this outcome was either not present or where there was little or inadequate detail to report on

N.B. A single review often reported on multiple outcomes (e.g. outcomes for anxiety and depression)

### Depression

Reviews reporting depression outcomes (*n* = 25) were published between 2018 and 2024 and were mainly systematic reviews (see Supplementary file 4 for full details). The number of studies included in reviews ranged from 7 to 50, with a total of 477 studies across reviews and an average of 19 studies. Of those reviews presenting on depression outcome findings, 11/25 reported improvement, 4/25 no significant change, and this was not clear in 10/25 reviews.

Where positive outcomes were reported, the focus of the interventions tended to be described (as stated in review aims) as psychosocial interventions (e.g. educational counselling), non-pharmacological interventions (*n* = 2) (e.g. grief therapy), psychoeducational interventions (e.g. caregiver self-caret) (*n* = 2), supportive interventions (e.g. skills development) (*n* = 2), or a combination of these terms (*n* = 2). One review focused exclusively on e-Health interventions [74]. Theoretical bases were reported for 3/11 reviews [49, 67, 79], which included coping and stress; illness-focused; cognitive, change theories; interpersonal/relationship focused; problem-solving/skills focused; other (e.g. grounded theory) bases. Where details were available, interventions were most frequently delivered online/web-based (*n* = 9) and via telephone (*n* = 9), to home (*n* = 5) and health facilities (*n* = 4), largely facilitated by nurses (*n* = 5), mental health professionals (*n* = 5), and social workers with four reviews including interventions which were wholly or partly self-guided.

### Anxiety

The evidence reporting on anxiety outcomes was published between 2014 and 2024 (*n* = 23), largely comprised of systematic reviews (21/23) (see Supplementary file 5 for full details). The number of studies included in reviews ranged from 2 to 50 (average 19), with a total of 440 included studies. In these reviews, nine reported improvements, five no significant change, and the findings were not clear in nine reviews.

In the nine reviews reporting favourable findings for improving carer anxiety, interventions of interest were described as inclusive of psychoeducational interventions (*n* = 3), non-pharmacological (*n* = 2), psychosocial (*n* = 1), and psychological (*n* = 1). Additionally, two reviews had a more specialised focus: art-based interventions [69] and web-based compassion interventions [88]. Four of the nine reviews reported on theoretical bases of included intervention studies [49, 67, 79, 80] which included frameworks from the following classifications: coping and stress; illness-focused; cognitive, change theories; interpersonal/relationship focused; problem-solving/skills focused; and other. Most frequently interventions were facilitated by mental health professionals (*n* = 5), followed by nurses (*n* = 4) and social workers (*n* = 4), with four reviews including interventions which were wholly or partly self-guided.

### Psychological distress

Reviews reporting on psychological distress were published between 2018 and 2023 (*n* = 14), and the majority were systematic reviews (13/14) (see Supplementary file 6 for details). The number of studies included in reviews ranged from 8 to 50, with a total of 325 studies across reviews and an average of 23 studies. Psychological distress was reported as improved in seven reviews, as no significant change in two and the findings were unclear in five reviews.

Of the reviews reporting positive outcomes, interventions of interest were described as including psychosocial interventions (*n* = 3), supportive interventions (*n* = 2), psychosocial and/or educational (*n* = 1), and one community-based cancer telephone services (*n* = 1). Three of these reviews reported on theoretical bases which were represented in the following classifications: coping and stress, illness-focused, cognitive, interpersonal/relationship focused, problem solving-skills focused, and other. Interventions were largely delivered online/web-based (*n* = 6) and via telephone (*n* = 6), into health facility (*n* = 5), and home (*n* = 4) settings. Most frequently these interventions were facilitated by health professionals, namely, nurses (*n* = 6) followed by mental health professionals (*n* = 3) and then followed by social workers (*n* = 2) and doctors (*n* = 2), with three reviews including interventions which were wholly or partly self-guided.

### Quality of life

Reviews reporting on quality of life were published between 2013 and 2024 (*n* = 27) and were almost all systematic reviews (26/27) (see Supplementary file 7 for details). Collectively 540 studies were included across reviews (ranging between 6 and 50), with an average of 20 studies. Quality of life was reported as improved in 12 studies, with no significant change in three and the findings were not clear in the remaining 12.

Reviews reporting improvements in this outcome identified interventions of interest in terms of psychoeducational interventions (*n* = 3), psychosocial interventions (*n* = 3), supportive interventions (*n* = 3) (of which two were focused on health technology), psychological intervention (e.g.) (*n* = 1). One review specifically examined peer-group interventions for children with parents with cancer [56] and another examined ‘mind–body therapies’ [78]. Six of these 12 reviews reported theoretical bases [14, 48, 49, 56, 63, 67], including coping and stress, change theories, illness-focused, cognitive theories, interpersonal/relationship focused, problem solving-skills focused, and other. Interventions were largely reported as delivered online/web-based (*n* = 9) and via telephone (*n* = 7), into health facility (*n* = 6) and home (*n* = 5) settings. Commonly interventions were facilitated by nurses (*n* = 5) and mental health professionals (*n* = 5) with most reviews included interventions which were wholly or partly self-guided (*n* = 7).

## Discussion

This study presents a mapping of the evidence about the psychosocial interventions available to promote the well-being of carers for people with cancer. In doing so, this work aligns with and helps to clarify the evidence supporting recommendations and standards of survivorship care which highlight the needs of carers and the interrelationship between the well-being of survivors and carers [15–17]. Despite the volume and upward trend in published reviews over the last decade, there is significant fragmentation of this evidence which means that building a foundation of knowledge that informs the development, delivery, and sustenance of supportive programmes for carers is challenging.

The lack of clarity regarding theoretical bases for interventions in the evidence, which here were under- and inconsistently reported, poses an impediment to building knowledge on existing evidence about what works and how [55, 74]. Cancer carer-specific theory (e.g. Fletcher’s conceptual model of the cancer family carer experience) [38] was, notably, not prevalent. In reviews reporting favourable outcomes, theories (where reported) centred on coping and stress and cognitive theories were most commonly represented. Overall, contributions of the review findings to theory building or confirmation were limited, highlighting the need for this reporting in future reviews.

Review evidence included a combination of in-person and remotely offered interventions, often delivered via telephone and internet/web-based, with a combination of live and pre-recorded modes too. Nurses and mental health professionals were the most frequently represented facilitators. Reviews reporting improvements in outcomes generally reflected these patterns of delivery modes and facilitators. Additionally, self-guidance in at least some aspect of interventions was apparent in over a third of reviews and, where reported, was identified in at least half of reviews indicating improved outcomes. This finding is perhaps unsurprising in light of advancements in digital health technology that increase capabilities to facilitate self-management [89]. Facilitation appears to be a key feature of interventions, with health professionals well-placed to be involved, and with opportunities arising for guided interventions in the digital health spaces.

Important issues and ambiguities concerning carer populations were identified. Most reviews concerned adult patient populations (as expected); however, a subset concerned paediatric patient populations [46, 53, 54, 66, 70, 77, 79, 83], highlighting the opportunity and need to explore evidence for specific carer cohorts. A core challenge in our search process was the large volume of reviews identified in which carers were not the sole or primary focus, but which did include some reference to carer participation and/or findings (often difficult to distinguish from the findings concerning other populations). A potential explanation is that interventions may be extended to include carers but may not necessarily have been designed for carers [25]. A related issue is that few reviews provided details of the processes of intervention development/adaptation and information about the involvement of carers and other stakeholder groups [42, 82, 85]. This raises concerns about how to ensure that programmes are designed to meet the specific needs of carers [24, 37]. Documentation of the involvement of carers and other key stakeholders in intervention development in primary studies and the reporting of these details in reviews would provide valuable learnings [24, 37]. In a recent review of consumer involvement and the application of co-design approaches in oncology studies, a paucity of dedicated reporting guidelines which can capture the involvement of consumers and stakeholders was observed to be a serious impediment [90]. We concur with the recommendation of the review about the value of developing dedicated reporting guidelines [90], which in turn, would, if widely-utilised aid the collection and reporting of this data in reviews.

A key limitation of the study design is that meta-reviews are subject to the quality of data and any flaws of the included reviews [32], with some concerns regarding aspects such as the implementation of methods to minimise errors in data collection in reviews identified via appraisal. Additionally, combining findings from reviews can be challenging owing to differences in populations, interventions, or outcome measures, rendering it difficult to form an overall conclusion [33]. Inconsistency and variation in the targeting and reporting of carer outcomes contribute to fragmentation of the evidence. This may offer an explanation for the instances in which review outcome findings were not clear enough to report on. Finally, the findings are restricted to literature published in English and evidence currently synthesised in reviews [91].

## Conclusion and future directions

A refocusing on clearly defined research is needed to advance the field. These efforts could be benefited by a greater focus on the unique needs of carers, more targeted interventions which focus on specific issues or specific carer population, more judicious use of outcome measures, and an enhanced focus on implementation of evidence into policy and practice, inclusive of identifying and addressing barriers to uptake and investment in implementation of effective interventions. Consistent reporting of core outcomes regarding carer cohorts supporting people with particular cancers, carer population, or period in the caring journey would enhance evidence synthesis efforts and facilitate larger scale studies that conduct subgroup analyses for different carer cohorts. A research agenda is needed which embeds theory-driven approaches, which would also require that future research document and report on the theoretical bases guiding interventions. Carer-specific programmes are needed with further reporting of how interventions have been designed to address the specific needs of a particular carer group. This meta-review is a useful first step to enabling this broad and often disconnected evidence base to be easier to navigate, engage with and use.

## Supplementary Information

Below is the link to the electronic supplementary material.

Supplementary file1 (DOCX 72 KB)

## Data Availability

No datasets were generated or analysed during the current study.
