# Supplementary material for: What can we learn from the evidence of psychosocial support for carers of people with cancer and how do we advance our efforts? A meta-review study
Source: J Cancer Surviv. 2025 May 16;20(Suppl 1):1155–76. doi: 10.1007/s11764-025-01802-8 (PMC13676645; doi:10.1007/s11764-025-01802-8)
Supplement: Supplementary file 1 [file 11764_2025_1802_MOESM1_ESM.docx]

# **Supplementary file 1:** Database(s): Ovid MEDLINE(R) ALL 2013 to February 03, 2023 Search Strategy

| **#** | **Searches** |
| --- | --- |
| 1 | exp neoplasms/ |
| 2 | (cancer* or oncology* or neoplasm* or carcinoma* or tumo?r* or malignan* or lymphoma* or melanoma* or leuk?emia or sarcoma).tw. |
| 3 | 1 or 2 |
| 4 | (family or families or parent* or mother? or father? or friend? or relative? or spous* or partner? or husband? or wife or wives or son? or daughter? or offIspring? or sibling? or brother? or sister? or informal or friends or significant other*).tw. and (care* or caring).mp. |
| 5 | caregivers/ or caregiver burden/ |
| 6 | (carer* or caregiv* or care giv*).tw. |
| 7 | exp home nursing/ |
| 8 | exp family/ |
| 9 | or/4-8 |
| 10 | ((family or families or parent* or mother? or father? or friend? or relative? or spous* or partner? or husband? or wife or wives or son? or daughter? or offspring? or sibling? or brother? or sister?) adj4 (support* or inform* or train* or educat* or teach* or coach* or instruct* or advis* or advice* or counsel* or therap* or cbt or program* or psycho* or social or pastoral or spiritual or religio* or self help or selfhelp)).mp. [mp=title, book title, abstract, original title, name of substance word, subject heading word, floating sub-heading word, keyword heading word, organism supplementary concept word, protocol supplementary concept word, rare disease supplementary concept word, unique identifier, synonyms] |
| 11 | problem solving.tw. |
| 12 | social support/ or psychosocial.ti,hw. or psychosocial intervention*.mp. or psychosocial support*.mp. |
| 13 | exp social work/ |
| 14 | exp psychotherapy/ |
| 15 | exp counseling/ |
| 16 | education/ |
| 17 | health education/ |
| 18 | teaching/ |
| 19 | exp "religion and psychology"/ |
| 20 | self help groups/ |
| 21 | self care/ |
| 22 | problem solving/ |
| 23 | professional family relations/ |
| 24 | ((psychosocial adj4 intervention*) or (psychosocial adj4 support*) or (support* adj4 intervention*)).ti. |
| 25 | or/10-24 |
| **26** | **3 and 9 and 25** |
| **27** | **limit 26 to (english language and yr="2013 -Current")** |
| 28 | (systematic review or meta-analysis).pt. |
| 29 | meta-analysis/ or systematic review/ or systematic reviews as topic/ or meta-analysis as topic/ or "meta analysis (topic)"/ or "systematic review (topic)"/ or exp technology assessment, biomedical/ or network meta-analysis/ |
| 30 | ((systematic* adj3 (review* or overview*)) or (methodologic* adj3 (review* or overview*))).ti,ab,kf. |
| 31 | ((quantitative adj3 (review* or overview* or synthes*)) or (research adj3 (integrati* or overview*))).ti,ab,kf. |
| 32 | ((integrative adj3 (review* or overview*)) or (collaborative adj3 (review* or overview*)) or (pool* adj3 analy*)).ti,ab,kf. |
| 33 | (data synthes* or data extraction* or data abstraction*).ti,ab,kf. |
| 34 | (handsearch* or hand search*).ti,ab,kf. |
| 35 | (mantel haenszel or peto or der simonian or dersimonian or fixed effect* or latin square*).ti,ab,kf. |
| 36 | (met analy* or metanaly* or technology assessment* or HTA or HTAs or technology overview* or technology appraisal*).ti,ab,kf. |
| 37 | (meta regression* or metaregression*).ti,ab,kf. |
| 38 | (meta-analy* or metaanaly* or systematic review* or biomedical technology assessment* or bio-medical technology assessment*).mp,hw. |
| 39 | (medline or cochrane or pubmed or medlars or embase or cinahl).ti,ab,hw. |
| 40 | (cochrane or (health adj2 technology assessment) or evidence report).jw. |
| 41 | (comparative adj3 (efficacy or effectiveness)).ti,ab,kf. |
| 42 | (outcomes research or relative effectiveness).ti,ab,kf. |
| 43 | ((indirect or indirect treatment or mixed-treatment or bayesian) adj3 comparison*).ti,ab,kf. |
| 44 | (multi* adj3 treatment adj3 comparison*).ti,ab,kf. |
| 45 | (mixed adj3 treatment adj3 (meta-analy* or metaanaly*)).ti,ab,kf. |
| 46 | umbrella review*.ti,ab,kf. |
| 47 | rapid review*.ti,ab,kf. |
| 48 | (multi* adj2 paramet* adj2 evidence adj2 synthesis).ti,ab,kf. |
| 49 | (multiparamet* adj2 evidence adj2 synthesis).ti,ab,kf. |
| 50 | (multi-paramet* adj2 evidence adj2 synthesis).ti,ab,kf. |
| 51 | (synthesis* adj3 evidence).ti,ab,kf. |
| 52 | or/28-51 |
| **53** | **27 and 52** |
| 54 | (developed countries or european union or oecd).tw,hw,sh. |
| 55 | europe/ or andorra/ or austria/ or belgium/ or exp france/ or exp germany/ or exp united kingdom/ or greece/ or ireland/ or exp italy/ or liechtenstein/ or luxembourg/ or monaco/ or netherlands/ or portugal/ or exp "scandinavian and nordic countries"/ or spain/ or switzerland/ or exp australia/ or new zealand/ |
| 56 | north america/ or exp canada/ or exp united states/ |
| 57 | (united kingdom or england or scotland or wales or denmark or finland or iceland or norway or sweden).tw,hw,sh. |
| 58 | (europe* or andorra or austria or belgium or france or germany or greece or ireland or italy or liechtenstein or luxembourg or monaco or netherlands or portugal or spain or switzerland or australia* or new zealand).tw,hw,sh. |
| 59 | or/54-58 |
| **60** | **53 and 59** |
| 61 | (developing countr* or third world or underdeveloped countr* or under developed countr*).mp. |
| 62 | exp africa/ or americas/ or exp caribbean region/ or exp central america/ or latin america/ or mexico/ or exp south america/ |
| 63 | exp europe, eastern/ or exp transcaucasia/ |
| 64 | antarctic regions/ or exp atlantic islands/ or exp indian ocean islands/ or exp pacific islands/ |
| 65 | New Guinea/ or asia/ or exp asia, central/ or asia, southeastern/ or borneo/ or cambodia/ or east timor/ or indonesia/ or laos/ or malaysia/ or mekong valley/ or myanmar/ or philippines/ or thailand/ or vietnam/ or asia, western/ or bangladesh/ or bhutan/ or india/ or middle east/ or afghanistan/ or iran/ or iraq/ or jordan/ or lebanon/ or oman/ or saudi arabia/ or syria/ or turkey/ or yemen/ or nepal/ or pakistan/ or sri lanka/ or far east/ or china/ or tibet/ or exp korea/ or mongolia/ |
| 66 | (Afghanistan or Africa or Albania or Algeria or Angola or Antigua or Argentina or Armenia or Azerbaijan or Bangladesh or Barbados or Barbuda or Belarus or Belize or Bhutan or Bolivia or Bosnia or Botswana or Bulgaria or Burkina Faso or Burundi or Cambodia or Cameroon or Central African Republic or Chad or Chile or china or Colombia or Comoros or Congo or Costa Rica or Croatia or Cuba or Czech* or Congo or Djibouti or Dominica or Dominican or East Timor or Ecuador or Egypt or El Salvador or Equatorial Guinea or Eritrea or Estonia or Ethiopia or Fiji or Gabon or Gambia or Ghana or Grenada or Guatemala or Guinea-Bissau or Guyana or Haiti or Honduras or Hungary or India or Indonesia or Iran or Iraq or Ivory Coast or Jamaica or Jordan or Kazakhstan or Kenya or Kiribati or Kyrgyzstan or Laos or Latvia or Lebanon or Lesotho or Liberia or Libya or Lithuania or Madagascar or Malawi or Malaysia or Maldives or Mali or Marshall Islands or Mauritania or Mauritius or Mexico or Micronesia or Moldova or Mongolia or Montenegro or Morocco or Mozambique or Myanmar or Namibia or Nepal or New Guinea or Nicaragua or Niger or Nigeria or Korea or Oman or Pakistan or Palau or Panama or Papua New Guinea or Paraguay or Benin or China or Peru or Philippines or Poland or Cape Verde or Georgia or Kosovo or Macedonia or Yemen or Romania or Russia or Rwanda or Saint Kitts or Saint Vincent or Saint Lucia or Sao Tome Principe or Saudi Arabia or Senegal or Serbia or Seychelles or Sierra Leone or Slovak* or South Africa or Solomon Islands or Somalia or Sri Lanka or Sri-Lanka or Sudan or Suriname or Swaziland or Syria or Tajikistan or Tanzania or Thailand or Togo or Tonga or Trinidad or Tobago or Tunisia or Turkey or Turkmenistan or Uganda or Ukraine or Uruguay or Uzbekistan or Vanuatu or Venezuela or Vietnam or Samoa or Zambia or Zimbabwe).af. |
| 67 | (low income countries or middle income countries).mp. [mp=title, book title, abstract, original title, name of substance word, subject heading word, floating sub-heading word, keyword heading word, organism supplementary concept word, protocol supplementary concept word, rare disease supplementary concept word, unique identifier, synonyms] |
| 68 | 66 or 67 |
| 69 | 53 not 68 |
| **70** | **60 or 69** |

**Supplementary file 2.** Summary of critical appraisal by quality criteria and number of studies.

| Quality criteria items | Yes | No | Unclear | N/A |
| --- | --- | --- | --- | --- |
| 1. Is the review question clearly and explicitly stated? | 54 |  |  |  |
| 2.Were the inclusion criteria appropriate for the review question? | 52 |  | 2 |  |
| 3. Was the search strategy appropriate? | 53 |  | 1 |  |
| 4.Were the sources and resources used to search for studies adequate? | 52 |  | 2 |  |
| 5.Were the criteria for appraising studies appropriate? | 38 |  | 11 | 5 |
| 6. Was critical appraisal conducted by two or more reviewers independently? | 24 | 1 | 24 | 5 |
| 7.Were there methods to minimize errors in data extraction? | 40 |  | 14 |  |
| 8.Were the methods used to combine studies appropriate? | 47 |  | 7 |  |
| 9.Was the likelihood of publication bias assessed? | 16 | 1 | 35 | 2 |
| 10.Were recommendations for policy and/or practice supported by the reported data? | 51 |  | 3 |  |
| 11. Were the specific directives for new research appropriate? | 54 |  |  |  |

# **Supplementary file 3:** Theoretical bases reported in review texts

| Citation | Coping and stress | Change theories | Illness-focused | Cognitive theories | Interpersonal/  relationship focused | Educational | Problem solving/  skills focused | Therapies | Other |
| --- | --- | --- | --- | --- | --- | --- | --- | --- | --- |
| Ahn et al. (2020) | ✓ |  |  | ✓ | ✓ |  |  |  | ✓ |
| Al Daken & Ahmad (2018) |  |  |  |  |  |  |  |  | ✓ |
| Becqué et al. (2023) | ✓ |  |  | ✓ | ✓ |  |  | ✓ | ✓ |
| Boele et al. (2019) | ✓ |  |  | ✓ |  |  |  |  |  |
| Cheng et al (2022) | ✓ | ✓ | ✓ | ✓ |  |  |  |  |  |
| Dhumal et al. (2023) | ✓ |  |  | ✓ |  |  |  | ✓ | ✓ |
| Eccleston et al. (2015). |  |  |  | ✓ | ✓ |  | ✓ | ✓ |  |
| Eche et al. (2021) | ✓ |  | ✓ | ✓ | ✓ |  | ✓ |  |  |
| Ferrell & Wittenberg (2017) |  |  |  |  | ✓ |  |  |  |  |
| Fu et al. (2017) | ✓ |  |  | ✓ | ✓ | ✓ |  | ✓ | ✓ |
| Gabriel et al. (2020) | ✓ |  |  | ✓ | ✓ |  |  |  |  |
| Geertz et al. (2023) | ✓ |  |  | ✓ | ✓ |  |  |  | ✓ |
| Kajiwara et al. (2023) | ✓ |  |  |  |  |  |  |  | ✓ |
| Kalyani et al. (2023) | ✓ |  |  |  |  |  |  |  |  |
| Kleine et al. (2019) |  | ✓ |  |  |  |  |  |  |  |
| Kusi et al. (2023) | ✓ | ✓ |  | ✓ | ✓ |  | ✓ |  | ✓ |
| Lally & Brooks (2016) | ✓ | ✓ |  | ✓ |  |  |  |  |  |
| Lehto et al., (2021) |  |  |  |  |  |  |  |  | ✓ |
| Murfield et al. (2019) |  |  |  |  | ✓ |  |  |  | ✓ |
| Petersen (2020) |  |  |  |  |  |  |  |  | ✓ |
| Sak-Dankosky et al. (2022) |  |  |  |  | ✓ |  |  |  |  |
| Sánchez-Egea et al. (2019) |  |  |  | ✓ |  | ✓ |  |  |  |
| Sutanto et al. (2021) | ✓ |  |  | ✓ |  |  |  |  |  |
| Tang et al. (2020) | ✓ |  | ✓ |  |  |  |  |  |  |
| Thomas et al. (2017) | ✓ |  |  | ✓ |  |  |  |  | ✓ |
| Treanor et al. (2019) | ✓ |  |  | ✓ | ✓ |  |  | ✓ | ✓ |
| Wootten et al. (2014) |  |  |  | ✓ | ✓ | ✓ | ✓ | ✓ |  |
| Yıldız et al. (2024) |  |  |  | ✓ |  |  |  |  | ✓ |
| Details for other included theories | Ahn et al. (2020): custom designed conceptual model; Al Daken & Ahmad (2018): Mindfulness; Becqué et al. (2023): conceptual model “Hanging on the Hope; conceptual model of Quality of Life,; adult teaching principles and the self-care concept, existential phenomenological therapy, research-derived, Calgary Family Assessment Model, Andershed & Ternestedt relatives involved in palliative care, preliminary live with love conceptual framework; Dhumal et al. (2023): Positive Psychology theory and family caregiver quality of life theory; Geertz et al. (2023): “…Clarke’s school-age child support group model; resource-oriented positive psychology; systemic solution focused therapy; recommendations of the international COSIP [Children of somatically ill parents] group for families with a somatically ill parent; intentional sequencing of activities and planned debriefing of activities are used for educational and therapeutic purpose; based on the premise that children are powerfully shaped by their external environment and behaviour is a function of social context, which has been referred to as the ‘power of context’; normalization, and expression; principles of mental health promotion; and Hult and Waads salutogenic, health promoting methodology; selfcompassion, continuing bonds, constructivism and meaning making approaches to grief were used in the development of the program.” (pp4-5); Kajiwara et al. (2023): Positive psychology; Kusi et al. (2023): Grounded theory ; Model of Family Caregiver Quality of Life; Theoretical framework of Andershed and Ternested; Van Houtven’s model; Lehto et al., (2021): theoretical underpinnings of natural environment approaches include: “the power of nature to rest and restore one’s directed attention capacity” and “mechanisms by which the natural environment has the capacity to reduce perceived stress” (p11124).; Murfield et al. (2019) “theory and learning from across evolutionary, developmental, social, and Buddhist psychology, as well as affective neuroscience, and highlights the importance of the three basic emotion-regulation systems within mental health” and “framework of dialectical philosophy and validation, which sees the manualised approach involve continual orchestration between change-orientated strategies and acceptance.” (p8); Petersen (2020): spirituality in supporting coping; Thomas et al. (2017): quality-of-life frameworks; Treanor et al. (2019): crisis intervention model (Morley et al.), equity theory; and Yıldız et al. (2024): Behavioral counseling theory, learning theories, Buddhist traditions, Meaning-centered psychotherapy model. | | | | | | | | |

# **Supplementary file 4: Overview of reviews reporting an outcome for depression**

| Citation | Review details | Study designs included | No. of included studies | Details of intervention population | Patient population: adult/ paediatric or both | Condition(s) of those cared for | Theoretical base groups represented | Details of intervention settings; modes of delivery and facilitation included in reviews | Depressive symptoms |
| --- | --- | --- | --- | --- | --- | --- | --- | --- | --- |
| Ahn et al., 2020 | Systematic review | RCTs and quasi-experimental designs | 11 | Family caregivers of adult patients with advanced cancer | Adult | Advanced cancer | Coping and stress; Cognitive theories; Interpersonal/ relationship focused; Other | Home and outpatient; remote and in-person; Nurses, doctors, interdisciplinary teams, mental health professionals | Unclear |
| Akechi et al., 2022 | Systematic review with meta-analysis | RCTs | 25 | Bereaved families of patients with a physical illness including cancer | Both | Physical illness including cancer | Not reported/ Unclear | Not reported/ Unclear | Improved |
| Cheng et al., 2022 | Systematic review with meta-analysis | RCTs | 12 | Adult caregivers caring for adult patients with cancer. | Adult | Cancer (any) | Coping and stress; Change theories; Illness-focused; Cognitive theories; | Settings not reported/ Unclear; remote and in-person; Nurses, psychologists, interventionists and multidisciplinary group. | Improved |
| Chow et al., 2023 | Systematic review  With meta-analysis | RCTs | 49 | Adult caregivers of adult patients with advanced (stage III or IV) cancer | Adult | Advanced cancer | Not reported/ Unclear | Outpatient, inpatient and home; mode not reported/ Unclear; Nurse, psychologist, social worker, self-guided, practitioner, multidisciplinary team | Improved |
| Dhumal et al., 2023 | Systematic review  with meta-analysis | RCTs | 11 | Adult ICs engaged in provision of adult cancer care | Adult | Cancer (any) | Coping and stress; Cognitive theories; Therapies; Other | Setting not reported/ unclear; mode not reported/unclear; nurse, researcher, peer support, doctor, social worker | No significant change |
| Frambes et al., 2018 | Systematic review | RCTs | 14 | Caregivers of people with cancer | Not specified/ Unclear | Cancer (any) | Not reported/ Unclear | Setting not reported/ unclear; remote and in-person; Facilitators not reported/ unclear; | Improved |
| Heinsch et al., 2022 | Systematic review | Quantitative, qualitative or mixed methods | 13 | Adult carer and patient cohort (>18 years) | Adult | Primary brain tumour | Not reported/ Unclear | Setting not reported/ unclear; remote and in-person; Electronic support network, nurse, specialists and website moderators cancer care coordinator | Unclear |
| Hughes et al., 2023 | Systematic review | No restrictions | 11 | Informal caregivers of adults with cancer | Adult | Cancer (any) | Not reported/ Unclear | Home, clinic; odes not reported/ unclear; nurse, self-guided, interdisciplinary team, psychologist. | Unclear |
| Koumarianou et al., 2021 | Systematic review | Randomized controlled trials and quasi-experimental studies | 17 | Families with children newly diagnosed aged between 7–12 years | Paediatric | Cancer (any) | Not reported/ Unclear | Home, clinic; remoted and in-person; nurses, psychologists, social worker, doctor and multidisciplinary team | Improved |
| Kusi et al., 2023 | Systematic review with meta-analysis | RCTs and non‐RCTs | 28 | Adult caregivers providing care to adult cancer patients | Adult | Cancer (any) | Coping and stress; Change theories; Cognitive theories; Interpersonal/ relationship focused; Problem solving/ skills focused; Other | Hospital, home; mode not reported/ unclear; bilingual-bicultural para-professionals; social worker, nurse, researcher, psychologist, oncologist, therapist, self-guided, oncology experts and priest | Improved |
| Lee et al., 2021 | Systematic review  with meta-analysis | RCTs | 15 | Informal caregivers for a persons with advanced cancer | Not specified/ Unclear | Advanced cancer | Not reported/ Unclear | Not reported/ Unclear | Improved |
| Lei et al., 2023 | Systematic review  with meta-analysis | Intervention studies | 8 | Lung cancer patients’ informal caregivers | Not specified/ Unclear | Lung cancer | Not reported/ Unclear | Home; mode not reported/ unclear; health care professionals | Improved |
| Li et al., 2022 | Systematic review  with meta-analysis | RCTs | 7 | Adult informal caregivers of patients with cancer | Adult | Cancer (any) | Not reported/ Unclear | Home; remote; self-guided | Improved |
| Liu et al., 2023 | Systematic review  with meta-analysis | RCTs and quasiexperimental studies | 8 | Primary caregivers of patients with breast cancer | Not specified/ Unclear | Breast cancer | Not reported/ Unclear | Not reported/ Unclear | Improved |
| Murfield et al., 2019 | Scoping review | Quantitative, qualitative, or mixed-method research designs | 32 | Adult carers of an older adult family member, friend, or neighbour. | Adult | Conditions impacting older people: | Interpersonal/ relationship focused; Other | Setting not reported/ unclear; remote and in-person; self-guided, therapists, social worker, researcher, psychologists and counsellor | Unclear |
| Roderick et al., 2024 | Systematic review | RCTs | 11 | Adult carers of individuals with cancer | Both | Cancer (any) | Not reported/ Unclear | Home, hospital; mode not reported/ unclear; self-guided (other facilitators but not identified). | Unclear |
| Sak-Dankosky et al., 2022 | Systematic review | RCTs and quasi-experimental studies | 27 | Caregivers of adult patients with cancer | Adult | Cancer (any) | Interpersonal/ relationship focused | Outpatient; remote and in-person; nurse, social worker, therapist, psychologists, health educator, artist, self-guided; mindfulness trainer and interdisciplinary teams. | Improved |
| Sánchez-Egea et al., 2019 | Systematic review  with meta-analysis | RCTs and uncontrolled trials | 40 | Family members of children with cancer | Paediatric | Cancer (any) | Cognitive theories; Educational | Not reported/ Unclear | Unclear |
| Secinti et al., 2022 | Systematic review  with meta-analysis | RCTs | 50 | Adult carers of adults with cancer - notable that 79% of carers were caucasian | Adult | Cancer (any) | Not reported/ Unclear | Not reported/ Unclear | Unclear |
| Shin et al., 2018 | Systematic review | No restrictions | 18 | Caregivers of patents with cancer | Not specified/ Unclear | Cancer (any) | Not reported/ Unclear | Home, hospital; remote and in-person; self-guided, nurse | Unclear |
| Tang et al., 2020 | Systematic review  with meta-analysis | RCTs | 11 | Caregivers of children (up to 18 years) undergoing treatment for cancer | Paediatric | Cancer (any) | Coping and stress; Illness-focused; | Home; remote and in-person; nurse, psychologist and facilitator with psychology background. | No significant change |
| Treanor et al. 2019 | Systematic review  with meta-analysis | RCTs | 19 | Adult non-professional caregiver of an adult patient with cancer | Adult | Cancer (any) | Coping and stress; Cognitive theories; Interpersonal/ relationship focused; Therapies; Other | Outpatient, home, residential setting; remote and in-person; nurses, psychologists, mental health specialists, therapists, social worker, study authors, multidisciplinary team, and other health professionals. | No significant change |
| Winter et al., 2023 | Systematic review | RCTs and pilot studies of interventions | 24 | Partners and informal carers of men with prostate cancer | Adult | Prostate cancer | Not reported/ Unclear | Home, other settings not reported; mode not reported/ unclear; facilitators not reported/ unclear; | Unclear |
| Yıldız et al., 2024 | Systematic review | RCTs | 8 | Family members caring for patients with cancer. | Not specified/ Unclear | Advanced cancer | Cognitive theories; Other | Home, private office, others not specified; mode not reported/ unclear; psychologist, social worker, psychological counselor interns, oncology clinicians, mindfulness teacher, self-guided and nurse | Unclear |
| Zhu et al., 2024 | Systematic review  with meta-analysis | RCTs | 8 | Informal caregivers aged 18 years; | Not specified/ Unclear | Any illness | Not reported/ Unclear | Setting not reported/ unclear; mode not reported/ unclear; self-guided, compassion practitioners, researchers, psychology students and mindfulness teachers | No significant change |

**Supplementary file 5: Overview of reviews reporting an outcome for anxiety**

| Citation | Review type | Study types/ designs included in review | Total no. of included studies | Details of intervention population | Patient population: adult/ child | Condition(s) of those cared for | Theoretical bases for interventions | Details of intervention settings; modes of delivery and facilitation included in reviews | Anxiety symptoms |
| --- | --- | --- | --- | --- | --- | --- | --- | --- | --- |
| Ahn et al., 2020 | Systematic review | RCTs and quasi-experimental designs | 11 | Family caregivers of adult patients with advanced cancer | Adult | Advanced cancer | Coping and stress; Cognitive theories; Interpersonal/ relationship focused; Other | Home and outpatient; remote and in-person; Nurses, doctors, interdisciplinary teams, mental health professionals | Unclear |
| Akechi et al., 2022 | Systematic review with meta-analysis | RCTs | 25 | Bereaved families of patients with a physical illness including cancer | Both | Physical illness including cancer | Not reported/ Unclear | Not reported/ Unclear | No significant change |
| Bautista et al., 2021 | Systematic review with meta-analysis | RCTs | 14 | Parents of children with cancer | Paediatric | Cancer (any) | Not reported/ Unclear | Settings not reported/ unclear; in-person; facilitators not reported/ unclear | No significant change |
| Cheng et al., 2022 | Systematic review with meta-analysis | RCTs | 12 | Adult caregivers caring for adult patients with cancer. | Adult | Cancer (any) | Coping and stress; Change theories; Illness-focused; Cognitive theories; | Settings not reported/ unclear; remote and in-person; Nurses, psychologists, interventionists and multidisciplinary group. | Improved |
| Chow et al., 2023 | Systematic review  With meta-analysis | RCTs | 49 | Adult caregivers of adult patients with advanced (stage III or IV) cancer | Adult | Advanced cancer | Not reported/ Unclear | Outpatient, inpatient and home; mode not reported/ Unclear; Nurse, psychologist, social worker, self-guided, practitioner, multidisciplinary team | Improved |
| Dhumal et al., 2023 | Systematic review  with meta-analysis | RCTs | 11 | Adult ICs engaged in provision of adult cancer care | Adult | Cancer (any) | Coping and stress; Cognitive theories; Therapies; Other | Setting not reported/ unclear; mode not reported/unclear; nurse, researcher, peer support, doctor, social worker | No significant change |
| Hughes et al., 2023 | Systematic review | No restrictions | 11 | Informal caregivers of adults with cancer | Adult | Cancer (any) | Not reported/ Unclear | Home, clinic; odes not reported/ unclear; nurse, self-guided, interdisciplinary team, psychologist. | Unclear |
| Kusi et al., 2023 | Systematic review with meta-analysis | RCTs and non‐RCTs | 28 | Adult caregivers providing care to adult cancer patients | Adult | Cancer (any) | Coping and stress; Change theories; Cognitive theories; Interpersonal/ relationship focused; Problem solving/ skills focused; Other | Hospital, home; mode not reported/ unclear; bilingual-bicultural para-professionals; social worker, nurse, researcher, psychologist, oncologist, therapist, self-guided, oncology experts and priest | Improved |
| Lally & Brooks, 2016 | Integrative review | RCTs and pre-post designs (including pilots). | 6 | Supporters of women with early stage breast cancer who were from a western culture | Adult | Breast cancer (early stage) | Coping and stress; Change theories; Cognitive theories; | Home, community; remote and in-person; nurse, therapist, counsellor, community health worker and educational counsellor | Unclear |
| Lang & Lim, 2014 | Systematic review with meta-analysis | Experimental study designs including quasi-experimental (pre post) | 2 | Adult family caregivers. | Not specified/ Unclear | Cancer (any) | Not reported/ Unclear | Setting not reported/ unclear; in-person; facilitator not reported/ unclear | Improved |
| Lee et al., 2021 | Systematic review with meta-analysis | RCTs | 15 | Informal caregivers for a persons with advanced cancer | Not specified/ Unclear | Advanced cancer | Not reported/ Unclear | Not reported/ Unclear | Unclear |
| Lei et al., 2023 | Systematic review  with meta-analysis | Intervention studies | 8 | Lung cancer patients’ informal caregivers | Not specified/ Unclear | Lung cancer | Not reported/ Unclear | Home; mode not reported/ unclear; health care professionals | Improved |
| Liu et al., 2023 | Systematic review  with meta-analysis | RCTs and quasiexperimental studies | 8 | Primary caregivers of patients with breast cancer | Not specified/ Unclear | Breast cancer | Not reported/ Unclear | Not reported/ Unclear | Improved |
| Murfield et al., 2019 | Scoping review | Quantitative, qualitative, or mixed-method research designs | 32 | Adult carers of an older adult family member, friend, or neighbour. | Adult | Conditions impacting older people: | Interpersonal/ relationship focused; Other | Setting not reported/ unclear; remote and in-person; self-guided, therapists, social worker, researcher, psychologists and counsellor | Unclear |
| Roderick et al., 2024 | Systematic review | RCTs | 11 | Adult carers of individuals with cancer | Both | Cancer (any) | Not reported/ Unclear | Home, hospital; mode not reported/ unclear; self-guided (other facilitators but not identified). | Unclear |
| Sak-Dankosky et al., 2022 | Systematic review | RCTs and quasi-experimental studies | 27 | Caregivers of adult patients with cancer | Adult | Cancer (any) | Interpersonal/ relationship focused | Outpatient; remote and in-person; nurse, social worker, therapist, psychologists, health educator, artist, self-guided; mindfulness trainer and interdisciplinary teams. | Improved |
| Sánchez-Egea et al., 2019 | Systematic review  with meta-analysis | RCTs and uncontrolled trials | 40 | Family members of children with cancer | Paediatric | Cancer (any) | Cognitive theories; Educational | Not reported/ Unclear | Improved |
| Secinti et al., 2022 | Systematic review  with meta-analysis | RCTs | 50 | Adult carers of adults with cancer - notable that 79% of carers were caucasian | Adult | Cancer (any) | Not reported/ Unclear | Not reported/ Unclear | Unclear |
| Shin et al., 2018 | Systematic review | No restrictions | 18 | Caregivers of patents with cancer | Not specified/ Unclear | Cancer (any) | Not reported/ Unclear | Home, hospital; remote and in-person; self-guided, nurse | Unclear |
| Tang et al., 2020 | Systematic review  with meta-analysis | RCTs | 11 | Caregivers of children (up to 18 years) undergoing treatment for cancer | Paediatric | Cancer (any) | Coping and stress; Illness-focused; | Home; remote and in-person; nurse, psychologist and facilitator with psychology background. | No significant change |
| Treanor et al. 2019 | Systematic review  with meta-analysis | RCTs | 19 | Adult non-professional caregiver of an adult patient with cancer | Adult | Cancer (any) | Coping and stress; Cognitive theories; Interpersonal/ relationship focused; Therapies; Other | Outpatient, home, residential setting; remote and in-person; nurses, psychologists, mental health specialists, therapists, social worker, study authors, multidisciplinary team, and other health professionals. | No significant change |
| Winter et al., 2023 | Systematic review | RCTs and pilot studies of interventions | 24 | Partners and informal carers of men with prostate cancer | Adult | Prostate cancer | Not reported/ Unclear | Home, other settings not reported; mode not reported/ unclear; facilitators not reported/ unclear; | Unclear |
| Zhu et al., 2024 | Systematic review  with meta-analysis | RCTs | 8 | Informal caregivers aged 18 years; | Not specified/ Unclear | Any illness | Not reported/ Unclear | Setting not reported/ unclear; mode not reported/ unclear; self-guided, compassion practitioners, researchers, psychology students and mindfulness teachers | No significant change |

**Supplementary file 6:** Overview of reviews reporting an outcome for psychological distress

| Citation | Review details | Study designs included | No. of included studies | Details of intervention population | Patient population: adult/ paediatric or both | Condition(s) of those cared for | Theoretical base groups represented | Details of intervention settings; modes of delivery and facilitation included in reviews | Depressive symptoms |
| --- | --- | --- | --- | --- | --- | --- | --- | --- | --- |
| Ahn et al., 2020 | Systematic review | RCTs and quasi-experimental designs | 11 | Family caregivers of adult patients with advanced cancer | Adult | Advanced cancer | Coping and stress; Cognitive theories; Interpersonal/ relationship focused; Other | Home and outpatient; remote and in-person; Nurses, doctors, interdisciplinary teams, mental health professionals | Improved |
| Becqué et al., 2023 | Systematic review | Quantitative studies | 11 | Adult family caregivers providing care to patients with advanced cancer | Paediatric | Advanced cancer | Coping and stress; Cognitive theories; Interpersonal/ relationship focused; Therapies; Other | Home, outpatient hospital setting, in‐patient hospital setting, palliative care unit, hospice, GP office; mode not reported/ unclear ;self-guided, nurse, researcher, GP, clinician, carer advisor, psychologist, health educator, psychology postgraduate students, palliative care team, social work, including multidisciplinary team, physician, priest, health care professionals and therapist. | Unclear |
| Boele et al., 2019 | Systematic review | RCTs | 11 | Adult caregivers (aged 18 years or older) for people with a brain or spinal cord tumour. | Adult | Neuro-oncology (over 20% of sample) | Coping and stress; Cognitive theories; | Medical centre, cancer centre, hospital and rehabilitation; remote and in-person; psychologists, nurse, social workers, self-guided | Unclear |
| Dhumal et al., 2023 | Systematic review  with meta-analysis | RCTs | 11 | Adult ICs engaged in provision of adult cancer care | Adult | Cancer (any) | Coping and stress; Cognitive theories; Therapies; Other | Setting not reported/ unclear; mode not reported/unclear; nurse, researcher, peer support, doctor, social worker | Improved |
| Eche et al., 2021 | Systematic review with meta-analysis | Prospective, quasi-experimental or experimental design | 17 | Parents of children with cancer | Paediatric | Cancer (any) | Coping and stress; Illness-focused; Cognitive theories; Interpersonal/ relationship focused; Problem solving/ skills focused | Clinic, hospital, home; remote and in-person; self-guided, trained animals, dog handler, nurse, | No significant change |
| Heckel et al., 2019 | Systematic review | No restrictions | 18 | Adult caregivers of adult cancer patients | Adult | Cancer (any) | Not reported/ Unclear | Community; remote; nurses | Improved |
| Hughes et al., 2023 | Systematic review | No restrictions | 11 | Informal caregivers of adults with cancer | Adult | Cancer (any) | Not reported/ Unclear | Home, clinic; odes not reported/ unclear; nurse, self-guided, interdisciplinary team, psychologist. | Improved |
| Koumarianou et al., 2021 | Systematic review | Randomized controlled trials and quasi-experimental studies | 17 | Families with children newly diagnosed aged between 7–12 years | Paediatric | Cancer (any) | Not reported/ Unclear | Home, clinic; remoted and in-person; nurses, psychologists, social worker, doctor and multidisciplinary team | Unclear |
| Murfield et al., 2019 | Scoping review | Quantitative, qualitative, or mixed-method research designs | 32 | Adult carers of an older adult family member, friend, or neighbour. | Adult | Conditions impacting older people: | Interpersonal/ relationship focused; Other | Setting not reported/ unclear; remote and in-person; self-guided, therapists, social worker, researcher, psychologists and counsellor | Improved |
| Sak-Dankosky et al., 2022 | Systematic review | RCTs and quasi-experimental studies | 27 | Caregivers of adult patients with cancer | Adult | Cancer (any) | Interpersonal/ relationship focused | Outpatient; remote and in-person; nurse, social worker, therapist, psychologists, health educator, artist, self-guided; mindfulness trainer and interdisciplinary teams. | Unclear |
| Secinti et al., 2022 | Systematic review  with meta-analysis | RCTs | 50 | Adult carers of adults with cancer - notable that 79% of carers were Caucasian | Adult | Cancer (any) | Not reported/ Unclear | Not reported/ Unclear | Unclear |
| Shin et al., 2018 | Systematic review | No restrictions | 18 | Caregivers of patents with cancer | Not specified/ Unclear | Cancer (any) | Not reported/ Unclear | Home, hospital; remote and in-person; self-guided, nurse | Improved |
| Treanor et al. 2019 | Systematic review  with meta-analysis | RCTs | 19 | Adult non-professional caregiver of an adult patient with cancer | Adult | Cancer (any) | Coping and stress; Cognitive theories; Interpersonal/ relationship focused; Therapies; Other | Outpatient, home, residential setting; remote and in-person; nurses, psychologists, mental health specialists, therapists, social worker, study authors, multidisciplinary team, and other health professionals. | Improved |
| Winter et al., 2023 | Systematic review | RCTs and pilot studies of interventions | 24 | Partners and informal carers of men with prostate cancer | Adult | Prostate cancer | Not reported/ Unclear | Home, other settings not reported; mode not reported/ unclear; facilitators not reported/ unclear; | No significant change |

**Supplementary file 7: Overview of reviews reporting an outcome for quality of life**

| Citation | Review details | Study designs included | No. of included studies | Details of intervention population | Patient population: adult/ paediatric or both | Condition(s) of those cared for | Theoretical base groups represented | Details of intervention settings; modes of delivery and facilitation included in reviews | Depressive symptoms |
| --- | --- | --- | --- | --- | --- | --- | --- | --- | --- |
| Ahn et al., 2020 | Systematic review | RCTs and quasi-experimental designs | 11 | Family caregivers of adult patients with advanced cancer | Adult | Advanced cancer | Coping and stress; Cognitive theories; Interpersonal/ relationship focused; Other | Home and outpatient; remote and in-person; Nurses, doctors, interdisciplinary teams, mental health professionals | Unclear |
| Akechi et al., 2022 | Systematic review with meta-analysis | RCTs | 25 | Bereaved families of patients with a physical illness including cancer | Both | Physical illness including cancer | Not reported/ Unclear | Not reported/ Unclear | No significant change |
| Bautista et al., 2021 | Systematic review with meta-analysis | RCTs | 14 | Parents of children with cancer | Paediatric | Cancer (any) | Not reported/ Unclear | Settings not reported/ unclear; in-person; facilitators not reported/ unclear | No significant change |
| Becqué et al., 2023 | Systematic review | Quantitative studies | 11 | Adult family caregivers providing care to patients with advanced cancer | Paediatric | Advanced cancer | Coping and stress; Cognitive theories; Interpersonal/ relationship focused; Therapies; Other | Home, outpatient hospital setting, in‐patient hospital setting, palliative care unit, hospice, GP office; mode not reported/ unclear ;self-guided, nurse, researcher, GP, clinician, carer advisor, psychologist, health educator, psychology postgraduate students, palliative care team, social work, including multidisciplinary team, physician, priest, health care professionals and therapist. | Unclear |
| Boele et al., 2019 | Systematic review | RCTs | 11 | Adult caregivers (aged 18 years or older) for people with a brain or spinal cord tumour. | Adult | Neuro-oncology (over 20% of sample) | Coping and stress; Cognitive theories; | Medical centre, cancer centre, hospital and rehabilitation; remote and in-person; psychologists, nurse, social workers, self-guided | Improved |
| Cheng et al., 2022 | Systematic review with meta-analysis | RCTs | 12 | Adult caregivers caring for adult patients with cancer. | Adult | Cancer (any) | Coping and stress; Change theories; Illness-focused; Cognitive theories; | Settings not reported/ Unclear; remote and in-person; Nurses, psychologists, interventionists and multidisciplinary group. | Improved |
| Chow et al., 2023 | Systematic review  With meta-analysis | RCTs | 49 | Adult caregivers of adult patients with advanced cancer | Adult | Advanced cancer | Not reported/ Unclear | Outpatient, inpatient and home; mode not reported/ Unclear; Nurse, psychologist, social worker, self-guided, practitioner, multidisciplinary team | Improved |
| Ferrell & Wittenberg, 2017 | Systematic review with meta-analysis | RCTs | 11 | Caregivers of adult patients with cancer. | Adult | Cancer (any) | Interpersonal/  relationship focused | Setting not reported/unclear; remote and in-person; nurse, specialist care provider, such as a behavioral therapist, psychologist, mental health professional and self-guided. | Improved |
| Frambes et al., 2018 | Systematic review | RCTs | 14 | Caregivers of people with cancer | Not specified/ Unclear | Cancer (any) | Not reported/ Unclear | Setting not reported/ unclear; remote and in-person; facilitators not reported/ unclear | Unclear |
| Gabriel et al., 2020 | Systematic review with meta-analysis | Intervention studies: Randomized and non-randomized | 11 | Adult people with cancer and their family caregivers | Adult | Cancer (any) | Coping and stress; Cognitive theories; Interpersonal/  relationship focused | Setting not reported/ unclear; remote and in-person; nurses, health team | Unclear |
| Geertz et al., 2023 | Systematic review with meta-analysis | Evaluation or intervention studies | 12 | Caregivers providing care to adult cancer patients | Adult | Cancer (any) | Coping and stress; Cognitive theories; Interpersonal/  relationship focused; Other | Camp site, cancer centre, graduate school, hospital, rehabilitation site.; remote and in-person; professional support group leaders, facilitators, and others not specified | Improved |
| Heinsch et al., 2022 | Systematic review | Quantitative, qualitative or mixed methods | 13 | Adult carer and patient cohort (>18 years) | Adult | Primary brain tumour | Not reported/ Unclear | Setting not reported/ unclear; remote and in-person; Electronic support network, nurse, specialists and website moderators cancer care coordinator | Unclear |
| Hughes et al., 2023 | Systematic review | No restrictions | 11 | Informal caregivers of adults with cancer | Adult | Cancer (any) | Not reported/ Unclear | Home, clinic; odes not reported/ unclear; nurse, self-guided, interdisciplinary team, psychologist. | Unclear |
| Kalyani et al., 2023 | Systematic review | RCTs | 14 | Caregivers providing care to adult cancer patients | Adult | Cancer (any) | Coping and stress | Setting not reported/ unclear; in-person; facilitator not reported/ unclear; | Improved |
| Kusi et al., 2023 | Systematic review with meta-analysis | RCTs and non‐RCTs | 28 | Adult caregivers providing care to adult cancer patients | Adult | Cancer (any) | Coping and stress; Change theories; Cognitive theories; Interpersonal/ relationship focused; Problem solving/ skills focused; Other | Hospital, home; mode not reported/ unclear; bilingual-bicultural para-professionals; social worker, nurse, researcher, psychologist, oncologist, therapist, self-guided, oncology experts and priest | Improved |
| Lee et al., 2021 | Systematic review  with meta-analysis | RCTs | 15 | Informal caregivers for a persons with advanced cancer | Not specified/ Unclear | Advanced cancer | Not reported/ Unclear | Not reported/ Unclear | Improved |
| Lei et al., 2023 | Systematic review  with meta-analysis | Intervention studies | 8 | Lung cancer patients’ informal caregivers | Not specified/ Unclear | Lung cancer | Not reported/ Unclear | Home; mode not reported/ unclear; health care professionals | Improved |
| Li et al., 2022 | Systematic review  with meta-analysis | RCTs | 7 | Adult informal caregivers of patients with cancer | Adult | Cancer (any) | Not reported/ Unclear | Home; remote; self-guided | Improved |
| Liu et al., 2023 | Systematic review  with meta-analysis | RCTs and quasiexperimental studies | 8 | Primary caregivers of patients with breast cancer | Not specified/ Unclear | Breast cancer | Not reported/ Unclear | Not reported/ Unclear | Improved |
| Murfield et al., 2019 | Scoping review | Quantitative, qualitative, or mixed-method research designs | 32 | Adult carers of an older adult family member, friend, or neighbour. | Adult | Conditions impacting older people: | Interpersonal/ relationship focused; Other | Setting not reported/ unclear; remote and in-person; self-guided, therapists, social worker, researcher, psychologists and counsellor | Unclear |
| Roderick et al., 2024 | Systematic review | RCTs | 11 | Adult carers of individuals with cancer | Both | Cancer (any) | Not reported/ Unclear | Home, hospital; mode not reported/ unclear; self-guided (other facilitators but not identified). | Improved |
| Sak-Dankosky et al., 2022 | Systematic review | RCTs and quasi-experimental studies | 27 | Caregivers of adult patients with cancer | Adult | Cancer (any) | Interpersonal/ relationship focused | Outpatient; remote and in-person; nurse, social worker, therapist, psychologists, health educator, artist, self-guided; mindfulness trainer and interdisciplinary teams. | Unclear |
| Sánchez-Egea et al., 2019 | Systematic review  with meta-analysis | RCTs and uncontrolled trials | 40 | Family members of children with cancer | Paediatric | Cancer (any) | Cognitive theories; Educational | Not reported/ Unclear | No significant change |
| Secinti et al., 2022 | Systematic review  with meta-analysis | RCTs | 50 | Adult carers of adults with cancer - notable that 79% of carers were Caucasian | Adult | Cancer (any) | Not reported/ Unclear | Not reported/ Unclear | Unclear |
| Shin et al., 2018 | Systematic review | No restrictions | 18 | Caregivers of patents with cancer | Not specified/ Unclear | Cancer (any) | Not reported/ Unclear | Home, hospital; remote and in-person; self-guided, nurse | Improved |
| Waldron et al., 2013 | Systematic review | RCTs | 49 | Cancer patient and caregiver | Not specified/ Unclear | Physical illness including cancer | Not reported/ Unclear | Home; mode not reported/unclear; nurses and psychologists | Unclear |
| Winter et al., 2023 | Systematic review | RCTs and pilot studies of interventions | 24 | Partners and informal carers of men with prostate cancer | Adult | Prostate cancer | Not reported/ Unclear | Home, other settings not reported; mode not reported/ unclear; facilitators not reported/ unclear; | Unclear |
| Yıldız et al., 2024 | Systematic review | RCTs | 8 | Family members caring for patients with cancer. | Not specified/ Unclear | Advanced cancer | Cognitive theories; Other | Home, private office, others not specified; mode not reported/ unclear; psychologist, social worker, psychological counselor interns, oncology clinicians, mindfulness teacher, self-guided and nurse | Unclear |
